# Repetition Is Important to Students and Their Understanding during Laboratory Courses That Include Research

**DOI:** 10.1128/jmbe.00158-21

**Published:** 2021-06-30

**Authors:** Benjamin L. Wiggins, Haley Sefi-Cyr, Leah S. Lily, Caroline L. Dahlberg

**Affiliations:** a University of Washington, Seattle, Washington, USA; b Western Washington University, Bellingham, Washington, USA

## Abstract

Course-based undergraduate research experiences (CUREs) provide students with opportunities for the same gains that apprenticed research with faculty members offers. As their popularity increases, it is important that critical elements of CUREs be supported by thoughtful design. Student experiences in CUREs can provide important insights into why CUREs are so effective. We present evidence from students who participated in CUREs at the introductory, intermediate, and advanced levels, as well as from graduate teaching assistants for an introductory lab course that included a CURE. Students and teaching assistants describe repetition as a valuable element in CUREs and other laboratory experiences. We used student work and open-ended interviews to identify which of five previously described elements of CUREs students found important. Because repetition was particularly salient, we characterized how students described repetition as they experienced it in courses that contained full-length CUREs or “micro-CUREs.” In prompted interviews, students described how repetition in CUREs provided cognitive (learning concepts) and practical (learning technical skills) value. Recent graduates who had participated in CUREs at each level of their biology education were particularly aware that they placed value on repetition and acknowledged it as motivational in their own learning. Many students described repetition in metacognitive terms, which also suggests that as students advance through laboratory and CURE curricula, their understanding of how repetition supports their learning becomes more sophisticated. Finally, we integrated student descriptions to suggest ways in which repetition can be designed into CUREs or other laboratory courses to support scientific learning and enhance students’ sense of scientific identity.

## INTRODUCTION

Students who participate in biology research show important gains in science identity and are more likely to remain in biological science (1–3). From the standpoint of equity and inclusion, adding authentic research experiences to coursework creates a setting for all students to experience inquiry and experimentation regardless of their background (4). Course-based undergraduate research experiences (CUREs) are educational interventions that provide students with benefits to similar to those that their peers may experience when conducting independent research (5). This coursework can therefore be as effective as an apprenticed research experience (6–8). CUREs have the potential to further democratize the beneficial outcome to students if they are designed to be easy to apply in less-resourced classrooms. However, successful laboratory course design requires the clearest possible understanding of how students experience the central features of CURE curricula.

Inquiry-based laboratories in STEM (science, technology, engineering, and math) courses beyond biology can improve student outcomes and have become central in designs for facilitating research-driven changes in biology, chemistry, and physics education (9–17). In particular, project-based lab curricula can promote meaningful engagement in scientific practices across disciplines, including in undergraduate courses across science disciplines (15, 18–22). The experiences of students who take part in biology CUREs, specifically, have been encapsulated in a conceptual framework that draws on the primary features of CUREs (23). These key features include scientific practices, collaboration, iteration, discovery, and relevant research (5). Well-designed CUREs can support student outcomes (including but not limited to self-efficacy, research skills, and navigating scientific obstacles) and improve the quality of data that student researchers produce (2, 23, 24). Thus, elements that support the primary features of CUREs should be included in CURE curricula to enhance student learning (25).

As research about CUREs progresses, we are learning more about why features improve student outcomes, under what conditions, and for which students. While the rationale among faculty for adopting a CURE curriculum may focus on the discovery and relevant research principles, students do not necessarily place high value on the prospect of publishing novel research despite its being a driving factor in faculty’s design of a CURE (2, 26–29). In contrast, recent modeling predicts that student feelings of ownership in laboratory courses are directly influenced by their perception of collaboration and iteration (28). In studies of physics laboratory courses, student ownership likewise hinged on struggle and collaboration, rather than an initial excitement about the research question (30). Additionally, students described troubleshooting and interpersonal activities (such as collaboration) as necessary and definitely a feature of experimental physics, respectively (31). *A Framework for K-12 Science Education* and *Next Generation Science Standards* (NGSS) also describe scientific and engineering practices that mirror the goals of CUREs but do not require novel discovery or relevant research *per se* (32, 33). For example, asking questions and defining problems, planning and carrying out investigations, analyzing and interpreting data, constructing explanations and designing solutions, and obtaining, evaluating, and communicating information can all be carried out in the absence of a novel research question. These practices are echoed in models for assessment at the university level in chemistry, physics, and biology curricula (34–43).

In primarily quantitative work, the complex practice of repetition is cited as a critical element of the scientific process and is reported by students to be important in their laboratory coursework (5, 28, 44). Understanding how students experience iteration in their own words is important for ensuring that CUREs can be transformative curricula for students, and it is this gap in the current state of research that can be productively filled by more qualitative investigation. Our work focuses on how iteration is experienced by students in CUREs at all levels of a biology department’s curriculum. The long-term goal of this strand of research is to assist course designers in taking up design elements that help a greater number and diversity of students, especially in situations where entire CURE structures may not be feasible.

This work is situated in the lived educational experiences of students at a primarily undergraduate institution (PUI). The PUI context is useful for investigating CURE dynamics due to the combination of lower overall resources compared to R1 universities, a sizable population of students who transfer from community colleges, and a large enough student body to allow sampling.

We used small-group interviews and analyses of student work to help us answer the following research question: how and under what conditions do students at different stages of their undergraduate careers experience repetition during laboratory courses? Students in introductory, intermediate, and advanced laboratory-based courses, with varying amounts of CURE or CURE-like structure, all reported repetition as a central feature of their learning. The results of our research suggest that at all levels, in either short CUREs (“micro-CUREs”) or full-length CUREs, students highly value repetition in their learning process.

## METHODS

**FIG 1:**
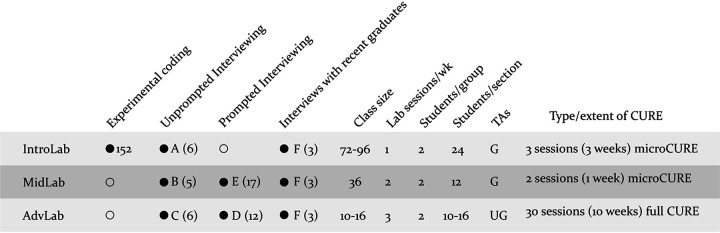
Overview of data collection for courses with CURE curricula. Data collection (experimental coding of student written work or interviews) is indicated for IntroLab, MidLab, and AdvLab. Letters next to filled circles refer to the interview groups described in Methods; numbers indicate the number of students interviewed for each course. Empty circles indicate that no data were collected.

Because the purpose of this research was to improve existing and ongoing education programs while simultaneously allowing the description of any generalizable findings, the flexible methodology of design-based research (DBR) was employed (45–48). DBR allows researchers to rigorously follow emergent understandings forward throughout mixed-method investigations. In order to determine which aspects of CURE courses were most important for students, we started by analyzing student work and conducting interviews with graduate teaching assistants and students. Classes varied in the amount of laboratory work that was part of the course. Students in introductory laboratory courses (IntroLab) randomly enrolled in either the traditional protocol-driven style laboratory course or an otherwise-identical course that had been redesigned with a three-session micro-CURE (49, 50). To follow up on our understanding of student experiences, we then directly interviewed students from the smaller mid-level laboratory (MidLab) and advanced laboratory (AdvLab) courses. In MidLab, a 1-week micro-CURE module was embedded within existing course content (51). AdvLab was run as a full-scale CURE. We interviewed students, teaching assistants, and one small group of students who had taken all three CURE/CURE-like courses. Figure 1 shows an overview of data collection. The methods and results below are presented in narrative form so that the reader can follow the progression of DBR investigation and subsequent adjustments in methods and data collection.

### Study population and context

Research was conducted at a mid-sized, public, primarily-undergraduate university (PUI) on courses run between autumn 2016 and autumn 2019. The student demographics for biology department majors for those years were 68% female, 17% students of color, and 29.5% first-generation college students; 45.5% of students had earned credits at a community college at the time of enrollment. These numbers are similar to those of the overall student body except for the percent females, which is higher than in the overall student body. Institutional review board (IRB) approval for this study was granted under exemptions EX16-084 (Western Washington University) and STUDY00002921 (University of Washington). All participating students were enrolled in biology courses that had CURE attributes (see Appendix S1 in the supplemental material), gave consent for every research element, and were in or pursuing STEM majors. Students were recruited quasirandomly by blind carbon-copied email to class lists for 1-h listening sessions with a response rate of 9 to 11%. Participants encompassed characteristics that qualitatively similar to those of the general student population in terms of ethnicity, race, gender, and first-generation college student status.

### Curricular context

Students were enrolled in cellular and molecular biology courses with varying CURE elements, all of which are 10-week courses. Appendix S1 contains a detailed description of the CURE elements in each course.

(i) For IntroLab (introductory cell and molecular biology), we designed a three-session micro-CURE that was introduced to one of two concurrent sections of the IntroLab course. Each course section met three times per week with a faculty instructor in the classroom and was subdivided into groups of 24 students who met together in the laboratory once per week. The laboratory sessions were led by graduate teaching assistants (GTAs). (ii) For MidLab (mid-level techniques-based molecular biology laboratory), a one-week micro-CURE was designed to deepen this technically-detailed but generally protocol-driven laboratory. Each course served up to 36 students, who attended once-weekly lectures led by the lab sections’ faculty instructors. The course was divided into three 12-person laboratory sections that met twice per week. (iii) AdvLab (senior-level advanced cell and molecular biology laboratory) is a full-length CURE course. This course was a 16-person laboratory course taught by a single faculty instructor.

### Initial investigations and coding

On course exams, students were given opportunities to provide written feedback regarding their introductory biology course. Our initial goal was to address the five core aspects of CURE design (as described in reference 5) through a series of interviews (Fig. 1). Students completed a traditional laboratory module (73 students) or a partially redesigned module in which 3 weeks had been devoted to a micro-CURE module (79 students). The prompt for student responses was, “How did this [laboratory] module change your understanding of how research is done?” For each cohort, transcripts of written responses were iteratively coded for evidence of five important CURE course themes. All data were confidential and anonymized during the research process. During the coding process, repetition emerged as a key component of student experiences that could differ between the treatment groups; further coding was team validated before analysis by comparing >10% of the coding set between all members of the research team.

### Interview data. (i) Open-ended qualitative interviews

We used interviews to capture important experiential elements of CURE modules from student experiences that did not fit into our known categories. Questions were written intentionally to allow broad responses without directed prompting; facilitators focused on creating an open atmosphere for honest feedback and probed students to explain their experiences more deeply and completely (52). For example, students in focus group B were asked broad prompts, including, “What is going well or poorly for you in your MidLab course?” and “Tell us about MidLab.” Recordings were used to create transcripts or develop deeper notes, as noted below. Interview sessions were 40 to 70 min in length.

The interview groups were as follows.

In focus group A, six GTAs who had worked in IntroLab were asked to describe their perceptions of undergraduate experiences in the course (not their own experiences as GTAs, as in reference 53). Emergent themes were described for analysis through interviewer notes and summaries. In focus group B, five undergraduate students currently in the second half of the quarter of MidLab were asked to describe their experiences in the course. Emergent themes were described for analysis through interviewer notes and summaries. In focus group C, six students who had recently completed AdvLab were asked to describe their experiences in the course. Emergent themes were described for analysis through interviewer notes and summaries. In focus group set D, 12 students who were taking AdvLab were interviewed 7 weeks into the 10-week quarter about their experiences. These transcripts were fully transcribed and coded for analysis; refer to Appendix S2 for the codebook.

### (ii) Prompted interviews

In order to more deeply elicit student experiences, further qualitative work specifically prompted students for these themes. This was an opportunity to ask how, for whom, and under what conditions particular elements of the CUREs were impactful for students. For example, an initial 10-minute open-ended discussion might be followed by interviewer prompts like, “You mentioned that some aspects of the course were significant to the experience; was repetition part of that?” Analyses of recorded transcripts from each session were used to assess student experiences around the primed aspect of conversation.

Interview groups were as follows.

In focus group and individual interview set E, eight total sessions were conducted with 17 students currently in the second half of the quarter of MidLab. These interviews and focus groups were fully transcribed and coded for analysis; refer to Appendix S3 for the codebook.

Interview set F involved longer interviews with three students who had taken all three courses. These interviews were fully transcribed and analyzed specifically for individuals’ discussions of repetition.

In total, interviews and focus groups summed to ∼50 h of student interview time and met our goals of broadly sampling the student population.

## RESULTS

### Results of initial investigations and coding

IntroLab students provided written answers to the question, “How did this [laboratory] module change your understanding of how research is done?” While we had originally been interested in descriptions of metacognition, student responses consistently conveyed the benefits of repetition. We coded the responses topically for the five primary features of CUREs (identified in reference 5), all of which are identified in Table 1. Note that a single student response could fit into multiple categories (85 of the 155 of students sampled identified 0 or 1 practices), but a student could code only for 1 or 0 in each category.

**TABLE 1:** Student reporting of five primary features of CUREs following laboratory modules^*a*^

Student group	No. (%) reporting:					
	Scientific practices	Collaboration	Discovery	Relevant research	Iteration	Total codes
CURE Lab	69 (86)	5 (6)	9 (11)	5 (6)	28 (35)	116
Traditional lab	66 (88)	15 (20)	7 (9)	8 (11)	19 (25)	115
All students	135 (87)	20 (13)	16 (10)	13 (8)	47 (30)	231

a Students in IntroLab gave written responses to the prompt, “How did this [laboratory] module change your understanding of how research is done?” Responses were coded using topically for five primary features of CUREs (5). The differences between the CURE and traditional labs were not statistically significant for any category (Student's *t* test, single-tailed; *P* > 0.05).

We expected to observe more student answers that fit into the scientific practices category because it is necessarily broader and also because this category is more central to the prompting question. Indeed, 87% of the codes fit into the scientific practices category. We were surprised to find that Iteration was the second most commonly coded category; we noted that students in the CURE course were more likely to respond by mentioning iteration (35%) versus students in the traditional lab (25%). This difference was not statistically significant (single-tailed, Student's *t* test) but suggests a trend in which students in authentic research experiences may perceive the impact of repetition as a useful part of science. All five primary features of CUREs represent areas for further analysis. However, we focused on repetition for deeper analysis for two reasons: (i) the unexpectedly large signal in mentions of iteration and (ii) the difference in mentions of iteration between the CURE and non-CURE treatment groups. We hope that understanding this importance of repetition in labs will help to identify ways to most beneficially employ repetition when designing CUREs.

### Results of interviews

We conducted a series of interviews and focus groups (see Methods). Coded transcripts revealed a series of related themes, which all reflected students’ understanding of repetition. These are summarized in Table 2. We discuss them in detail in the following two sections.

**TABLE 2:** Overview of repetition-related themes from student interviews^*a*^

Theme type	Theme identified
Affective	A lack of opportunities to repeat techniques prevented deeper learning
	Repetition instills confidence
	Repetition drives individual learning

Content	Repetition helps in learning lab techniques
	Conceptual topics are reinforced through lab repetition
	Repetition is an integral part of scientific research

a Themes were identified from interviews with GTAs from IntroLab and students from MidLab and AdvLab. Codebooks are available in the supplemental material.

### Open-ended inquiries into student experiences

As part of the curriculum development for the IntroLab CURE, we interviewed GTAs about their perceptions of students’ experiences in IntroLab (focus group A). Three core themes emerged from this focus group; repetition was the most prominent. (The two other emergent themes were length of the CURE and the value of student choice in laboratory decisions.) GTAs noted that students often linked repetition with productive failure, deliberate practice of techniques, and preparation for perceived high-stakes assessments. The descriptions of repetition from the GTA perspective directed us to interview undergraduates about their own perceptions of the CUREs in laboratory course settings. Because the IntroLab CURE was not offered annually, we interviewed students at intermediate and advanced levels of the curriculum.

In open-ended interviews with students who were finishing MidLab (focus group B) the strongest theme was again repetition. Students in MidLab felt “dropped into the deep end” of complicated lab techniques but unanimously recognized large progress in their own learning. As one student stated near the end of the course, “The first 2 weeks were impossible…now that stuff is just stupidly simple.” The specific catalyst for this change was the course design that connected individual lab sessions with opportunities to practice similar skills. For example, a lab that taught a particular technique (PCR) was followed by a lab that required successful PCR to complete a subsequent lab task. Repetition during and between labs facilitated a final lab session in which students revisited an experiment of their choice. This session provided no new content from instructors, but for some students it was the source of the greatest perceived learning because they recognized their own progress from low to high independence. Learning during MidLab included gains in metacognitive skills: the repetitive nature of the course “…gave me the skill of being OK with being stupid for a while and knowing that I will work out of that struggle period.” Without prompting, students said that opportunities for repetition provided them with an understanding of their own learning gains. MidLab represents a clear transition between introductory and advanced laboratory content and practice, so we next interviewed students in the AdvLab CURE.

When we interviewed senior-level students who had recently completed AdvLab (focus group C), they reported that repetition had the greatest impact on their learning and perception of other CURE principles. They described repetition as “failure and misery…this is what actual research is like, what you have to endure,” which indicated that this student saw failure as having a career-relevant impact. Students reported that repetitive lab exercises increased confidence in their own grasp of scientific material and helped them realize their newfound expertise. They also frequently described this metacognitive achievement as “troubleshooting.” Without prompting for repetition, advanced students reported that repetition and especially troubleshooting were core to the benefits of their CURE experience; this suggested that we might be able to more fully understand these benefits by following up on impromptu discussions of repetition during the interviews.

In a later iteration of AdvLab, another group of students were interviewed about their experiences in the CURE (focus group D). To ensure that initial responses were unprompted, and to draw out other themes, we asked neutral follow-up questions. Advanced students expressed that repetition of lab tasks was integral to understanding and that the lack of opportunities to repeat techniques prevented deeper learning. As one student shared, “[A]s we go on to the quarter we’ve already done like three Western blots and I can already, just I can feel more confident with what I'm doing with those Western blots and I think with the PCR that we did learn in [IntroLab] but it was still pretty hit-or-miss, still learning how to do it, you know? I will always mess up one thing and it’ll always be a different thing each time but like, this time around just like ‘oh right, you know I forgot, this has to be done otherwise this thing will happen’.”

Going beyond lab techniques, these students also expressed the importance of lab repetition for cementing conceptual learning. When discussing learning in molecular and cellular biology in lecture-based courses, one student said, “I think during it I was pretty confused because it was …my first upper division class [and] it was short … so I think during it, yeah, my head was exploding and I, like, didn't completely comprehend everything I was doing at the moment. And so a lot of the stuff I learned was definitely … in the lab afterwards when I was doing it over and over again, had to, like, troubleshoot it and problem-solve… [it is] really helpful for … understanding what the process was that we did and what happened during it because you can troubleshoot it then you know what happened.”

Repetition helps students better understand the conceptual aspects of the scientific material and what physically and technically takes place in the laboratory experiments.

### Prompted interviews

Our findings from unprompted interviews suggested that repetition was very important to students during laboratory courses. We wanted to learn more about how students view the benefits of repetition for learning in these molecular/cellular laboratory courses. In order to specifically focus on repetition in our interviews, we prompted students to discuss the values or drawbacks of spending time repeating lab techniques in intermediate and advanced labs that had CURE components.

MidLab students (in interview and focus group set E) raised the following four themes in these interviews. First, they indicated that repetition allowed for better learning of lab techniques. Second, conceptual topics from lecture sessions were better and more concretely understood through lab repetition. This was voiced by the student who said, “There's stuff we were doing in our lab where, like, we're trying to isolate certain bacteria and then sequence them to see what they are…now I know how to sequence them and I know how to use these databases and all kinds of resources that just we've talked about and now we've actually done.”

These first two themes further supported our findings from unprompted interviews: students recognized that their own learning benefits from the opportunity to repeat in a laboratory. Third, students frequently indicated that repetition brought improvements in confidence: “I was tempted to just, like, try to reproduce data…but I was too tempted by the idea of trying to synthesize a novel exploration and just really like get in and see what problems I would run into…what I'm doing kind of feels like a playground at this point.”

Lastly, students were able to use opportunities for repetition to drive their own learning. One student indicated that “we are going back and we're taking an experiment that we've done and…redo it and get better results or answer a different question and I think that was really cool.”

Students at the intermediate level found cognitive and practical value in repetition as they moved between inquiry-based and research-based labs.

Current students at all levels described repetition as critical to their learning, so we were interested in how the experiences of postgraduation seniors who had been in CUREs were impacted by repetition in laboratory experiences. In interview set F, we prompted recent graduates (within 6 months of graduation) about their CURE experiences; we followed with specific prompts for ideas about the drawbacks and values of repetition. They expressed that repetition is an integral part of scientific research, exemplified by a recent graduate stating, “Actually, one of the experiments I’m running right now is doing a very similar experiment because we got contradictory results compared to what we would have thought based on that other paper. So we’re repeating some of that other experiment with our materials, to see if we get the same result we got before, or maybe we just did something technically different that changed our results…. Especially now that there’s more papers coming out every day, the same experiment could be people doing it in different labs, like independent studies kinda proving the same thing.”

These graduates demonstrated an awareness of the value and motivational role of repetition. An interviewee noted that, “Repetition is very, very important, very powerful. It gets you more confident in your techniques, and your thoughts about those techniques and your reasoning for them and it’s important to solidify things…[without doing so] it’s still like you’re not really applying it as much or I don’t care I got it wrong, in my head I’ll do it right the next time, like maybe…but do you actually…?”

Taken together, our data suggest that students deeply value the practice of repetition in lab courses, particularly in the context of CUREs. This was true regardless of the precise qualitative research method or course types, suggesting that the value in repetitive opportunities may be particularly generalizable and helpful in broader science learning contexts. Because we observed a pattern of metacognitive processes being described as troubleshooting and repetition, we have included a glossary of terms in student voice that were directly applicable to metacognition (Appendix S4).

## DISCUSSION

We investigated the use of repetition in STEM courses using DBR methodology through qualitative analysis of student experiences in CUREs and micro-CUREs at all levels of a molecular and cellular biology curriculum. While students described other principles of CUREs as well, we were struck by the continual reflections on repetition from students and their TAs at all curricular levels. We followed up by asking specifically about what makes repetition useful to students. They reported that repetition was helpful for the mastery of lab techniques, improved their confidence in science abilities, bettered their understanding of conceptual science, and gave opportunities to self-design their learning beyond the designed curriculum. This was true for students at all levels and was also true for some students in course sections that included no or minimal CURE components (control sections of IntroLab and MidLab, respectively).

Our results from students of CUREs and micro-CURE courses at a primarily undergraduate institution (PUI) suggest that iteration and productive failure are immediately important to students’ approaches to research. The importance of undergraduate researchers in carrying out faculty-driven research also provides a motivation for faculty to implement CUREs at a PUI (8). Our findings that students are motivated by their chances to repeat activities during CUREs will be important in revising future courses to best serve students as they become proficient researchers.

This research context and design methodology builds on earlier, fundamental work in CUREs. Previous research by used quantitative measures to survey a larger number of students in a CURE program for first-year students and identified positive impacts of discovery, collaboration, and repetition (28). Our work attempts to deepen this understanding by triangulating from a qualitative lens. Instead of asking students to weigh discrete choices, we explored their complex spoken responses and highlighted repetition as a practice that was repeatedly impactful throughout the curriculum. Our focus on these aspects of student learning required more intensive student participation in our research, especially notable in the interview structure. This resulted in smaller samples sizes than what might be expected for survey responses, for example. Nonetheless, these numbers are in line with other generalizable research on student experiences (2, 54).

While students are motivated by laboratory work in CUREs, this motivation is not solely from “real” or “authentic” research. In fact, the importance of novel investigation and publication is less motivational to students than originally expected (2, 27–29, 55). At the same time, one motivator for faculty running a CURE is likely the laboratory data that students generate, and repetition and reproducibility are important for faculty who want to publish the data generated in CUREs. Designing CUREs and CURE-like activities that facilitate productivity by including repetition is an important goal for faculty who might otherwise be uninterested or unable to approach a novel laboratory course design (8, 56).

When students reported on repetition, they often described it in terms of troubleshooting: making multiple attempts at the same technique, skill, or concept as a practice of uncovering their errors and iteratively improving (2, 23, 44). As described previously, this kind of failure was most useful when grades were not penalized (28, 57). Advanced students who participated in CURE-like activities described increased metacognitive gains when given opportunities to follow failures with repetition. While students rarely described metacognition in the formal terms of “thinking about their thinking,” troubleshooting requires self-reflection and redirection, both of which are hallmarks of metacognition (54). We suggest that one major value of repetition in laboratory courses, particularly CUREs, is an improvement in metacognition, even if students fail to fully recognize it. Indeed, in previous work, we found that students were unaware of their demonstrable gains in problem solving skills after a CURE (49). CUREs may aid in metacognition by providing space for students to self-reflect and redirect in a motivated context.

Of the five previously identified parts of CUREs (scientific practices, collaboration, iteration, discovery, and relevant research) (5), repetition is relatively easy to include in a course. Without designing additional modules, instructors can provide opportunities to use the same materials and equipment for students to redo laboratory work and expect productive cognitive practice. As they generate expertise, students can gradually approach mastery by progressing through increasingly relevant levels of legitimate peripheral participation (58), so full-length CUREs may not be required to achieve the gains from CURE curricula. The benefits to student learning are many and are perhaps most inexpensively added simply by removing some material and replacing it with options for productive, deliberate-practice-like repetition. Where feasible, this may broaden the scope for students who can take advantage of important positive impacts identified in CUREs in institutional contexts where CUREs are not feasible. In Table 3, we provide examples of ways that intentional repetition can be built into laboratory and CURE curricula. These examples, developed and tested within a PUI environment, are likely to be broadly applicable even in situations where funding, staffing, or expertise to carry out a CURE does not exist.

**TABLE 3:** Examples of how repetition can be built into laboratory and CURE curricula^*a*^

Repetition to build into course design	Relationship to scientific practice	Relationship to student experience
Practice/dry run before running lab experiment	Error and anomaly are normal parts of science; refining practice is ongoing	Build confidence in their skills before being able to trust their results
Activities that span the gap between lecture and laboratory	Technical mastery of lab skills can be achieved independently of underlying conceptual understanding, and repetition across spaces helps to practice these working skills	Students see value in lab skills relevant to careers in research, even if those skills are detached from conceptual knowledge
Understanding laboratory space	Confident use of a general lab space is gained through repeated use of multiple examples of different labs	Students need orientation to basic expectations, components, and norms of any lab, plus that specific lab space
“Repeat-a-lab” opportunities at the end of the term	Identifying the next step in an ongoing research program is key for career scientists who go beyond episodic lab exercises	Students metacognitively self-identify experiments or skills on which additional practice will help their learning

a Suggestions of ways in which instructors can build repetition into laboratory courses are described in terms of how they related to scientific practices (central column) and how students describe their efficacy in the laboratory.

These benefits may best be conceptualized within the framework of deliberate practice (59). Lab-based repetition bears the hallmarks of this productive set of practices; students use large amounts of time on task to attempt increasingly career-relevant challenges while receiving and acting on critical feedback. Deliberate practice is used widely across fields to understand best practices for skill development, and it is not surprising that students report similar benefits within their own undergraduate requirements. In the future, studies that follow student experiences after several years on the job market postgraduation will allow researchers and instructors to better understand which practices are the most helpful in the long-term. CUREs and other inquiry-based laboratory courses represent valuable opportunities for students. They recognize that their technical skills and content knowledge develop through repetition that is driven by the iterative nature of research. Designing laboratory courses with space for repetition will be an important way to support students as they mature from novice to expert scientists.
