# Supplementary material for: Repetition Is Important to Students and Their Understanding during Laboratory Courses That Include Research
Source: J Microbiol Biol Educ. 2021 Jun 30;22(2):e00158-21. doi: 10.1128/jmbe.00158-21 (PMC8442015; doi:10.1128/jmbe.00158-21)
Supplement: SUPPLEMENTAL FILE 1 — Download JMBE00158-21_Supp_1_seq2.docx, DOCX file, 0.03 MB [file jmbe00158-21_supp_1_seq2.docx]

Appendix 1.**Descriptions of CURE and “micro-CURE” elements in the courses featured in this study.**

*IntroLab:*

The three session micro-CURE on cell cycle control in Tetrahymena had a focus on metacognition for some sections of this course^1^. Students used GTA-presentations and bioinformatics analysis to help them generate hypotheses; they also evaluated the utility of different experimental approaches in testing their hypotheses, planned experiments, analyzed data, and actively reflected as they designed experiments and drew conclusions. The CURE ran for three of the nine total laboratory sessions of this ten-week course.

*MidLab:*

The one-week (two class meetings) micro-CURE for this course, which includes principles of gene expression and genetic modification, is part of an ongoing collaboration with nearby University of British Columbia. This collaboration required that students develop technical skills in PCR genotyping over the first eight weeks of the quarter. During the CURE week students are asked to genotype *C. elegans* that were engineered as part of a gene-editing initiative^2^. Students communicated their results to the collaborators^3^. The CURE ran for two of the eighteen total laboratory sessions of this ten-week course.

*AdvLab:*

This course is a full-length CURE wherein students choose from a set of related, but independent, research problems focused on protein quality control in *C. elegans*. Students perform genetic crosses, formalize rationales, design experiments, learn and perform pertinent techniques, deliver group meetings, and present at a poster session. The CURE ran for the entire ten-week course.

(1) Dahlberg, C. (Lina) L.; Lee, S. R.; Wiggins, B. L. A Short Laboratory Module to Help Infuse Metacognition during an Introductory Course-Based Research Experience. *CS* **2019**, *6*. https://doi.org/10.24918/cs.2019.20.

(2) Au, V.; Li-Leger, E.; Raymant, G.; Flibotte, S.; Chen, G.; Martin, K.; Fernando, L.; Doell, C.; Rosell, F. I.; Wang, S.; Edgley, M. L.; Rougvie, A. E.; Hutter, H.; Moerman, D. G. CRISPR/Cas9 Methodology for the Generation of Knockout Deletions in Caenorhabditis Elegans. *G3: Genes, Genomes, Genetics* **2019**, *9* (1), 135–144. https://doi.org/10.1534/g3.118.200778.

(3) Dahlberg, L.; Groat Carmona, A. M. CRISPR-Cas Technology In and Out of the Classroom. *The CRISPR Journal* **2018**, *1* (2), 107–114. https://doi.org/10.1089/crispr.2018.0007.

Appendix 2: Codebook for Interview Set D

Interviews with current AdvLab students about their experiences in MidLab (Set D). Students in this interview were not all enrolled in the same MidLab course. Quotes are labeled Adv.#, where # refers to the de-identified individual.

| Code | Definition | Notes | Quotes |
| --- | --- | --- | --- |
| Troubleshooting/problem solving | Dealing with when things go wrong or are surprising, and/or anticipating what unexpected things might mean | Used for Set E first | · Still learning how to do it, you know I will always mess up one thing and it’ll always be a different thing each time but like, this time around just like “oh right, you know I forgot, this has to be done otherwise this thing will happen.” Adv.2  · Existing troubleshooting as in “we plated bacteria and it didn't grow up” or it grew up too much o­­r whatever and then you're saying “okay well, we kind of have an idea of what's going on with it” by saying “did somebody add ampicillin to this” so there's already like known solutions of what probably went wrong versus if it's your own research then you are truly really delving into, like, what all the possibilities are or trying to figure out what other controls or what other experiments you can do to try to figure out what went wrong. Adv.3 |
| Persistence | Students showing persistence, or lack of persistence (giving up) | Generalized, includes reasons and motivations to persist or not | · It was overwhelming: the expectations before class and in class because it's something that no other lab class or anything like we've done before, like…Reading the protocol and writing it down and knowing what to do in lab is like such a big thing to do and then when you're in lab it's like you're trying to actually just complete something that you don't really understand yet. Adv.3  · I think that having a goal in general like makes you more motivated to finish something instead of just having a class that’s just like this week we’re doing this and this week we're doing this, just having one thing that you're excited about. Adv.5 |
| Validity/  relevance | Similar to “authenticity”-the work is “real” (e.g. CURE); or the experimental question is genuine; or the activity is seen as important to the future (e.g. “we need to know this if we want to do lab research”) | Parsed this into more specific codes but they seem to be very interlinked for students. For future: longer interviews with more room to probe could tease them out. | · There's less looking into it afterwards so if you put gfp into an organism and you're like cool my bacteria glows green but there's no follow-up of like what are we going to look for using that gfp so it's like very skills-based I feel instead of--which I think is the point cuz that class is skills-based this one is more applications based. Adv.1  · In MidLab it felt more like they’re throwing different like procedures at you to learn them and to know what they are, which is interesting but it wasn't-- it didn't all correlate and make sense to me as one big like study I guess, whereas over here it's like you know what the goal is that you're working towards and you have to come up with new techniques or new ways to go about it. Adv.5 |
| Iteration | Repetition, familiarity (or lack thereof), practice | Initially used only in a research context, then expanded because students brought it up outside that context | · I just want to be able to have more practice doing things like more pcr's and stuff like that and I knew this class did western Blots and I hadn't had an opportunity to do that here yet. Adv.1  · I feel like having repetition in those procedures is good because if they try to divvy up oh like last time we did this and this time I'll do that or whatever but I feel like it's a lot--a lot of times it's pretty easy to be like you did that last time you want to just do this and I'll do that cuz you're already familiar with it. Adv.2  · that's like your first time so you’re really just in the water and like splashing around trying to figure everything out, like, as it's happening. Adv.3 |
| collaboration | Working with other people or integrating other peoples’ work (e.g. literature search) | Future: Could parse it as something like “working on the same task together” vs “independent parts of a group endeavor” | · I know another goal in the course is to design—or like have you work with other people and collaborative skills but it's hard to do that and learned everything because you can't divide and conquer experiments because you want to learn how to do everything and so... I'm not sure that something that-- and it's not necessarily a big deal at least I got a little bit of both like learning how to collaborate and how to do lab techniques but it wasn't until AdvLab that because I had MidLab and I learned lab techniques then in AdvLab I could start to collaborate more. Adv.2  · You partnered up with somebody and so in that way you're learning collaborative skills with like a partner because you're working on the same project which is different from like AdvLab where people are working on different projects but you can still collaborate, and so like I knew I was collaborating in MidLab but I didn’t--it feels different from, you know, real scientific collaboration. Adv.2  · Especially undergrads who've never had a job before don't realize that the teacher and your employer are people and things do get messed up that frequently and so having AdvLab is a really great platform to move into a lab tech job or anything else cuz you just don’t realize until you have that experience that—because you’re gonna go wrong, and half the time you're going to spend figuring out why. Which like was a little bit of the case in MidLab but in AdvLab its pretty much like knowledge that you're going to be troubleshooting for yourself. Adv.4 |

Appendix 3. Codes for Interview/Focus Group Set E.

Students were interviewed about their experiences in MidLab. All students were participants in the same MidLab course. Quotes are labeled Mid.#, where # refers to the de-identified individual.

| **Code** | **Definition** | **Discussion** | **Quotes** |
| --- | --- | --- | --- |
| Grades and Teaching/Learning | Having to do with points, grades, course logistics, course setup | This code is probably not going to be helpful for analysis, but was included because it came up so much from the students. Current understanding is that this is typical (concern/anxiety over grades distracts students above all else). | · She's one of those professors that like I'll ask her a question and she'll answer with a question which is quite frustrating but it's helpful and it kind of and it makes me think you know and she asks very guided and pointed questions and that kind of leads me to come to the conclusion on my own a little bit. Mid.3  · I think that if the lecture were able to spend a little more time talking about the deeper conceptual plans or the idea behind it like what our learning objective is, then maybe it would be a little bit better but I think the lab a lot of what it does is just reiterate what our lab notebooks says--or sorry, the lecture, what the lecture does is reiterate what our lab notebook says.Mid.4  · it was so helpful, right, kind of consolidated what we had just done during the day and made you really think about in a quick period of time without a lot of pressure as far as points were concerned like if you got up and you said this is what my experiment was, this is what I think it means… you got points, which is amazing. Mid.5  · I think the lab could have been laid out clearer but I think the reason it wasn’t clearer was because they really wanted us to think about like why we were doing what we were doing which I appreciate in the end but sometimes in lab it would be like a little stressful. Mid.7 |
| Enjoyment | mention of "fun," "interesting," "cool," "exciting" | Notable due to its influence on motivation/engagement, and came up often around some of the nonstandard lab practices (as well as some of the standard ones) | · There’s been a lot of times where I'm like rushing through like making PCRs or something because we spend so much of that time figuring out what we're going to do, like all the math and stuff? that I can't even like enjoy myself pipetting. Mid.2  · None of it's relevant to what I wanted to do in the future but, BUT it's still just really really interesting like learning about restriction digests, dilutions are kind of annoying and suck but they're necessary part of learning how to do all that stuff. Mid.5 |
| External Trust | Students perceive that the expectations of the quality/success of their work are high. This is related to confidence in that external trust can inform/influence self-confidence but the core of this code is that there is another entity counting on (this would be "authenticity") or otherwise planning for/expecting success (or coping) | "Authenticity" was originally used, as an umbrella for "motivations to do the work or do it well that are not related to grades." This might become a separate code. "External trust" is used to reflect that students noticed and responded to expectations that they would/could handle a task on their own, regardless of consequences of completing or not completing the task | · In my chemistry labs they just give you like instructions and then you just do what it says but for this class specifically it gives you a lot of Independence so you have to like figure out what concentrations you need and all those things which were--which was frustrating at times just because the lab manual would be super vague, like “figure this out” and no one would really explain like how to figure it out. Mid.2  · We are involved in the research in that way but we're not directly performing like knocking out the gene. I guess we're just trying to make sure that what the other lab did worked, so we're just kind of looking at their results so I guess that's what I meant we weren't inserting the crispr and knocking out the genes we were seeing if the genes were knocked out. Mid.3 |
| Techniques and Concepts/Understanding | Specific techniques, or general mention of techniques; references to course content and understanding of it (often in contrast to the techniqutes practiced/learned | If this is an area of interest, this should probably become two codes in future work e.g. "Technique vs understanding" and "Integrated technique and content" | · I was asking myself simple questions in this class like what is a promoter, you know? Maybe I should have known that before so there was a lot of just trying to teach myself stuff that I should have already known. Mid.2  · Without having a fundamental understanding of how transcription works it's really difficult to go into that exam and say very confidently like “I understand what I did in this lab” and the reason behind that I think is that the labs are a lot of practice and they’re a lot of math so it's very hands-on…But the lecture portion really just introduces the lab as this is what you're going to be doing. Mid.4  · The lab manual is written like it's a light switch and all you need to do is turn it on and you just need like step one flip light switch up the lab manual’s like this is how electricity works. Mid.5 |
| Troubleshooting/Problem Solving | Dealing with when things go wrong or are surprising, and/or anticipating what unexpected things might mean | Students usually said "Troubleshooting", and used it to mean anticipating what could happen and understanding why it could happen. For precision, differentiate d"Troubleshooting" as being before a problem/result and "problem solving" being after it happens. Bigger picture metacognition might relate this to "Research and Science Expectations". For future: is there a difference in before/after? Or anticipating problems? (because students showed that they often expected things to go "wrong" and then maybe deal with them differently depending on the context of the experiment/task) | · You go step-by-step and then you ask yourself why does it look like this and if I wasn't expecting this then what went wrong and then if that went wrong could this result explain it you know, like if you have just a random band in the lane of a gel you could think “oh this could be contamination but what else could also cause that” cuz if you always just say it's contamination you're not going to get anywhere you could also could say like oh my PCR wasn't optimized correctly or something like that. You kind of have to just go 360 with how the results are shown. Mid.2  · Even before lab even before we got our results a lot of the time we would say well what could this mean what could this mean if we see these things. So I feel like I could do analysis. Mid.3  · You have what you would expect to have happen and you basically think about that and plan that out ahead of time and then something else happens and there's not a key that says oh if you get X result this is what it means, or if you get Y result this is what it means. You have to conceptually think about, well I had you know a band that was supposed to appear at 1800 and instead I have two bands one that appeared at 700 and one at 500, what does that mean? So yeah to me that's troubleshooting, and there's a lot of that in this course and in research in general. Mid.5 |
| Research and Science Expectations | How students conceive of the "real world" of scientific research or applications. Includes what skills will be necessary and/or useful, what norms or mores exist in the fields, and what it's like doing that kind of work (e.g. habits of mind, routines, expectations/requirements by supervisors/others) | This includes "soft" skills like working as a group, working towards a specific goal, interpersonal communication | · What really excites me about science is there's just like always new things and it's just like unimaginable. Cuz like even now, 30 years from now there's going to be something that comes up that we have no idea about right now.Mid.3  · It's the same pieces, we're just applying them in different ways to get ourselves some different information it seems like a lot of the same basic tools which I'm actually surprised by that so much of biology boils down to a very narrow range of tools or at least that's the impression I've gotten so far in my education. Mid.6 |
| Resources and Opportunities | Nonrequired activities done by students or offered to students (or not done, or not offered) | e.g. work-study research positions, office hours, online tutorials. See discussion of "self-confidence and persistence"--might need to be expanded to show lack of opportunities e.g. "couldn't register for the class" or "had to buy x software" | · My professor doesn't go over certain things other professors do, so I feel like some students have an advantage over me just because they get more material or like worksheets. Mid.2  · They might be like assuming that we have more skills to do things really quickly then we actually do like some of us still don't know how to use Excel that well and that takes a really long time but it's just kind of assumed “oh you have great knowledge of how to use Excel and you can graph something in 2 minutes”…and there’s no prereq from what I can tell that like teaches us Excel for this class. Mid.5 |
| confidence and persistence | Students showing, or lacking, confidence and persistence relating to their performance | "Stress" might also belong here as an opposite to self-confidence. This code, or perhaps "Resources and Opportunities," or both, might need to be delineated so that negative factors can be studied e.g. imposter syndrome, learned helplessness. | · It’s just kind of disheartening to get like 70% on something when I'm learning to use it for the first time. Mid.1  · That's always a nice feeling when she asks me a question and I'm like oh I do know this I just wasn't thinking about it in the right way and so I think that's--I've kind of gained that a little bit and that's been building throughout my whole academic career here but just kind of having confidence in that I've learned it and I know it and I just might not be thinking about it in the same way--in the like correct way that's going to lead me to where I'm trying to go. Mid.3  · You can't be discouraged yet you have to be able to step back and look through what went wrong and how you can make it better and I think just doing science as a general like feeling this class helped me to understand that stuff’s gonna go wrong and you got to be okay with that. Mid.6 |

Appendix 4: Glossary of terms relevant to repetition/iteration in laboratory courses from student voices. These show that repetition is a complex concept in terms of student language and that researching aspects of repetition in student experiences requires a broader lens of search terms. The third, fourth and fifth column display the results of an initial coding for complexity in regards to a prior hypothesis that student language about repetition may increase in complexity as students proceed through their degree program.

| Student Population: | Wording found in transcripts that is relevant to repetition/iteration: | Coded as: 'Relatively simple and not metacognitive' | Coded as related to: 'troubleshooting': | Coded as: 'Explicitly metacognitive' |
| --- | --- | --- | --- | --- |
| IntroLab | *An experiment should be repeatable* | 1 |  |  |
| IntroLab | *Importance of multiple trials* | 1 |  |  |
| IntroLab | *Important to do multiple runs/trials* |  |  |  |
| IntroLab | *Many more replicates need to be done* | 1 |  |  |
| IntroLab | *Many trials are important* | 1 |  |  |
| IntroLab | *Multiple trials are important* |  |  |  |
| IntroLab | *One trial/replicate doesn’t…prove results* |  |  |  |
| IntroLab | *Only through replication* | 1 |  |  |
| IntroLab | *Process of trial and error* | 1 |  |  |
| IntroLab | *Redo an experiment* | 1 |  |  |
| IntroLab | *Repeatable data* | 1 |  |  |
| IntroLab | *Repeating an experiment* | 1 |  |  |
| IntroLab | *Repeating experiments is very important* | 1 |  |  |
| IntroLab | *Replicated by other scientists* |  | 1 |  |
| IntroLab | *The importance of a dry run* | 1 |  |  |
| IntroLab | *The importance of repeating trials* | 1 |  |  |
| IntroLab | *The importance of repetition* | 1 |  |  |
| IntroLab | *Through many trials* |  |  |  |
| IntroLab | *Trial and error…must be repeated* |  | 1 |  |
| IntroLab | *Try things a few times* | 1 |  |  |
| IntroLab | *You don’t get it done one time and call it good* |  | 1 |  |
| IntroLab | *Controls and repetition are important* | 1 |  |  |
| IntroLab | *Finish the lab then do it again* | 1 |  |  |
| IntroLab | *Importance of practice trials* | 1 |  |  |
| IntroLab | *Importance of repeating experiments* |  |  |  |
| IntroLab | *Labs [need] multiple experiments* | 1 |  |  |
| IntroLab | *Many different experiments must be run* |  | 1 |  |
| IntroLab | *Multiple runs of an experiment is best* | 1 |  |  |
| IntroLab | *Multiple trials* |  |  |  |
| IntroLab | *Research requires multiple trials* | 1 |  |  |
| IntroLab | *Research should be reproduceable* | 1 |  |  |
| IntroLab | *Research should be…reproduceable* |  |  |  |
| IntroLab | *Running many trials* |  |  |  |
| IntroLab | *Supported data requires numerous tests* | 1 |  |  |
| IntroLab | *Supposed to be reproduceable* | 1 |  |  |
| IntroLab | *Trial and error…redoing trials* |  | 1 |  |
| MidLab | *[The lecture] just reiterates what our lab notebook says...lecture is kind of arbitrary…* |  |  |  |
| MidLab | *You’ll come back to things that you’ve learned in the past* |  |  |  |
| MidLab | *Ask him to explain it to me…go through power points* |  |  |  |
| MidLab | *Consolidated what we had just done* |  | 1 |  |
| MidLab | *Different ways of explaining things until I got it* |  |  |  |
| MidLab | *Getting ready for the experiments like reading through the manual and just setting up the notebook* |  | 1 |  |
| MidLab | *Go back to an old experiment and see if something didn’t go like we expected...talk through it* |  |  | 1 |
| MidLab | *Going back...redo it and get better results* |  | 1 |  |
| MidLab | *Going over and over and over again* | 1 |  |  |
| MidLab | *Good to look back on labs...we were looking at labs I want to do again so I’ll go back to the discussion...reflect back on later* |  |  |  |
| MidLab | *Hard to introduce somebody to something like that but now…* |  |  |  |
| MidLab | *Having the lab after is really good though because I feel like last week was just a dry run* |  | 1 |  |
| MidLab | *I can go back later and go okay what happened when I did that* |  | 1 |  |
| MidLab | *I did this before like I remember that when you flip back through your notebook and then I go like how did I calculate that* |  | 1 |  |
| MidLab | *I read the book and he...took the book and he’s like “here read this you’ll understand it”* |  |  |  |
| MidLab | *I watched it a couple times* |  |  |  |
| MidLab | *I watched it three times* | 1 |  |  |
| MidLab | *I’m actually going to be working on that next quarter so it was like directly applicable...it was a really great practice experience for me* |  |  | 1 |
| MidLab | *I’ve been prepping these labs for 4 years* |  |  |  |
| MidLab | *I’ve gotten a lot better...I got way more comfortable with the micropipetters* |  |  | 1 |
| MidLab | *If we had been able to practice like maybe a little bit more* |  |  |  |
| MidLab | *It took me a long time and so if it’s your first time...* |  |  |  |
| MidLab | *It's nice to have [the lab procedure] right there so I can like find it later on so I can see how it like came to be* |  |  | 1 |
| MidLab | *Kind of reiterating them* |  |  |  |
| MidLab | *Learning to use it for the first time* |  |  |  |
| MidLab | *Let’s go back and make sure everybody understands* |  | 1 |  |
| MidLab | *Listen in on lectures…a pre dose for the lab* |  |  |  |
| MidLab | *Most of the labs we’re doing the same thing...purpose would be the same like you want the same, like, understanding* |  |  |  |
| MidLab | *Most of the stuff we’re doing if just like exercises and illustrations anyway* | 1 |  |  |
| MidLab | *Practice with everything pretty consistently...different ways to do the same thing* |  | 1 |  |
| MidLab | *Rather than just having to copy everything down* |  |  |  |
| MidLab | *Really just a replication thing...the grunt work almost* |  |  |  |
| MidLab | *Redo things...decided to reproduce* |  |  |  |
| MidLab | *Redoing it with one variable changed* |  | 1 |  |
| MidLab | *Rerun an experiment and do everything the same and maybe change that one thing* |  | 1 |  |
| MidLab | *Same type of explanation in the class that she then used later in office hours...just a little bit more individualized* |  |  |  |
| MidLab | *Spend all this time out of class catching up* |  |  |  |
| MidLab | *Stuff that we practiced so many times* |  | 1 |  |
| MidLab | *Stuff we were doing in our lab...and now we’ve actually done* |  | 1 |  |
| MidLab | *Talk about it again...rerunning in the experiment* | 1 |  |  |
| MidLab | *The week 3 going back you don’t really have the fundamentals yet so like I'd push that back* |  |  | 1 |
| MidLab | *Useful to have all the practice* |  | 1 |  |
| MidLab | *We did it again today in like an hour flat so one you’ve done it once it just goes so much smoother but we didn’t get any practice for that before so I feel like a dry run would be really helpful* |  |  | 1 |
| MidLab | *We got to go back and do any lab that we wanted* |  |  |  |
| MidLab | *What went wrong and how do I fix it next time* |  | 1 |  |
| MidLab | *Yeah I’ve done this before, exactly, or like I’ve set up this lab for 3 months* |  | 1 |  |
| AdvLab | *Definitely like in the lab afterwards when I was doing it over and over again, had to like troubleshoot it and problem-solve...talked about what happened* |  |  | 1 |
| AdvLab | *Everyone is doing the exact same project* | 1 |  |  |
| AdvLab | *First doing that* |  |  |  |
| AdvLab | *Half of MidLabis PCR* | 1 |  |  |
| AdvLab | *I did cloning for like a whole year in her lab...exactly what I used in lab but just in general those skills are very helpful for other classes I’ve taken too* |  |  | 1 |
| AdvLab | *I did it and that’s all I really care about and then you walk away from it* | 1 |  |  |
| AdvLab | *I feel like having repetition in those procedures is good...last time we did this and this time I’ll do that...you did that last time you want to do this and I’ll do that 'cause you’re already familiar with it* |  |  | 1 |
| AdvLab | *I’d say at least for me personally it’s more like repetition...we’ve already done like three...I will always mess up one thing and it’ll always be a different thing each time but like, this time around* |  |  | 1 |
| AdvLab | *Just repetition more repetition I should say* | 1 |  |  |
| AdvLab | *Kind of repetitive* | 1 |  |  |
| AdvLab | *Learn skills like try to apply towards anything else* |  |  |  |
| AdvLab | *Make sure that we know all the techniques that you can use in case you want to do research later* |  |  | 1 |
| AdvLab | *Make sure that you can repeat stuff* |  | 1 |  |
| AdvLab | *More practice doing things...more experiences* | 1 |  |  |
| AdvLab | *One of the first classes I took at an upper division level* |  |  |  |
| AdvLab | *People have used this in the past...or are planning to in the future* |  |  | 1 |
| AdvLab | *Practicing the skills that you familiarized yourself with in 3MidLab* |  |  | 1 |
| AdvLab | *That's like your first time* | 1 |  |  |
